# Mutation-Based Harmony Search Algorithm for Hybrid Testing of Web Service Composition

**DOI:** 10.1155/2018/4759405

**Published:** 2018-11-01

**Authors:** Eckwijai Maythaisong, Wararat Songpan

**Affiliations:** Department of Computer Science, Faculty of Science, Khon Kaen University, Khon Kaen, Thailand

## Abstract

Web service composition is a method of developing a new web service from an existing one based on business goals. Web services selected for composition should provide accurate operational results and reliable applications. However, most alternative service providers have not yet fulfilled users' needs in terms of services and processes. Service providers, in fact, have focused on enhancing nonfunctional attributes, such as efficiencies of time, cost, and availability, which still face limitations. Furthermore, it remains advantageous to compose services and suitably plan them around business plans. Thus, this study introduces hybrid testing using a combination of the functional and nonfunctional testing approaches. The former was used to design a test case through the equivalence class partitioning technique, and the latter was used to select suitable services for the test results. We find defects and appropriate solutions for combining services based on business requirements. The mutation-based harmony search (MBHS) algorithm is proposed to select web services and to compose with minimum defects. The results of this study reveal that MBHS can support a combination of various services more efficiently and dramatically than other metaheuristic methodologies. Additionally, it helps find appropriate solutions to compose services based on business plans.

## 1. Introduction

The service-oriented architecture (SOA) concept is used to design organisational information systems as evolving service-oriented systems. The use of web services implies a SOA developing methodology [1]. SOA has been rapidly implemented to develop information systems because it encourages interoperability, considering changes in information, public relations, purchases, and services. Thus, web services can dramatically enhance business activities. Currently, various types of web services can be developed via simple object access protocols or representational state transfers [2]. With increases in system complexity, single web service mechanisms cannot respond to full system operations because data processing requires web services from other resources to be composed under the same conditions to achieve desired results.

Web service composition ensures that a web service concurs suitably with business goals. However, user needs and web services have grown more complex. Thus, web services will perform according to users' needs if they interoperate suitably with other web services. This is a primary advantage of web service composition [3]. Currently, the web services business process execution language is the standard for describing web service interoperations [4]. Furthermore, web service composition requires efficiency and quality. Various relevant studies have performed web service composition, focusing on quality of service (QoS) [5–7].

Web services have been widely used, both inside and outside organisations. The accuracy of service operational results is important. Generally, the problem of selecting a web service is the QoS and efficiency of its nonfunctional properties. Furthermore, during web service composition, it may never be reviewed if it provides operational results per the planned business service requirements. Additionally, any problems will appear as data defects. Functional testing is thus used to detect the nature of defects. In [8, 9], researchers implemented functional testing to detect minimum defects, and the results were as expected.

Currently, metaheuristic methodologies are used to estimate the fitness value of a web service composition for overcoming the complexity problem and reducing execution time. However, the primary purpose of metaheuristic development is to achieve optimisation. A web service developer anticipates the quality of optimisation, and metaheuristic algorithms are usually compared to the web service process and time constraints to identify the most efficient solution. In [10], the researcher categorised metaheuristic algorithms into groups to achieve optimisation.

Several familiar evolutionary algorithms are the genetic algorithm (GA) [11], genetic programming (GP), and differential evolution (DE). Swarm intelligence algorithms include the ant colony optimisation (ACO) and particle swarm optimisation (PSO) [12, 13]. Physics-related algorithms are simulated annealing (SA) [14] and harmony search (HS) [15, 16]. Harmony search (HS) algorithm is an interesting evolutionary algorithm which was developed in an analogy with music improvisation process, to improve the pitches of instruments and obtain better harmony. Whereas HS algorithm is good at identifying high performance of search space, when compared with other evolutionary algorithms [17], and it has still a drawback: the fixed parameter adjusting pitch adjusting rate (PAR) when applied the web service composition [18]. Adjusting pitch adjusting rate (PAR) is a very important factor for the high efficiency of the HS algorithm and useful for optimal search [19–22].

This study contributed to the current understanding of the innovation process in two main stages: (1) a hybrid testing approach for web service composition is combining the functional and nonfunctional testing approaches. The hybrid testing method is selected because it can estimate the minimum defect, the efficiency of the web service, and its fitness value and time cost when more web service providers are selected. (2) The new proposed metaheuristic algorithm is a mutation-based harmony search (MBHS). Optimisation is compared with GA, which is categorised by evolutionary algorithms, SA (physics-related) and PSO (swarm intelligence). Moreover, business models are also considered for optimisation of web service compositions. The proposed algorithm provides useful data for an efficient search algorithm and will estimate the minimum defects per business plans and user needs. The remainder of this study is organised as follows: Section 2 presents related works.  Section 3 presents the proposed framework. Experiments and evaluations are presented in Section 4. Finally, the conclusion and future works are presented in Section 5.

## 2. Related Work

The objective of testing a web service is to provide a system that operates well per the business goals. In [23], researchers designed criteria for evaluating a SOA to facilitate service providers and users and to develop a system resulting from commissioning, efficiency, and QoS criteria. The study of [6] designed characteristics of QoSs for SOAs to investigate the extent to which they affect the service of each characteristic and to accordingly adjust as the business requirements. Moreover, the technique used to check the validity of the web service has changed from document analysis to test case study. In [24], Bai et al. created a test case with the web services description language (WSDL), considering data types. Additionally, Hanna and Munro and Ma et al. [25, 26] implemented the boundary value analysis from WSDL to create a test case for testing services. In [27], Bhat and Quadri compared this to the equivalence class partitioning technique. However, this method retained limitations on efficiency checking.

As for the problems of enhancing the efficiency of selecting a web service with different types and components [4], most studies focused on QoS. For example, in [28], Sun and Zhao solved the problem of selecting and sorting web service QoSs in terms of cost, time, and reliability by using global and local QoSs. Moreover, in [29], researchers used GA to examine the best web service from global and local optimisations. Liu et al. [30] proposed a web service composition that sorted global and local optimisations through a cultural genetic algorithm per the QoS of each selected service, including execution time. Additionally, Wang et al. [31] proposed a web service composition evaluated by QoS attributes to suggest the best web service composition. Decision makers generally examine a web service based on its fitness for responding to a service delivery requirement. The study of Upadhyaya et al. [7] proposed a method for selecting a hybrid web service that found a compromise between QoS and user perceptions to maximise business requirements. Moreover, in [32], Lin et al. classified human and web services of different operations for efficiency.

Considering web service composition via metaheuristic fitness algorithms, the studies of Mardukhi et al. and Liu et al. [29, 30] applied genetic algorithms to find optimisation. Additionally, Fan et al. [33] implemented a stochastic swarm particle optimisation and simulated annealing to handle problems of selecting a web service composition based on QoS. The study of Parejo et al. [34] used GRASP and the path relinking hybrid algorithm to evaluate web service selection based on execution time. In [35], Yu et al. proposed service components for selecting efficient services from groups by using the greedy algorithm and ACO. These algorithms helped efficiently locate each group of complex services by time and quality. The study of Mao et al. [36] predicted the priority of QoSs for service providers and users using PSO, helping achieve optimisation and adjust to users' need for equivalence. In [37], the researchers implemented GP to estimate and analyse QoS. Moreover, Liu et al. [38] implemented social learning optimisation to enhance the efficiency of problem solving for selecting web services based on optimisation.

Currently, enhancing the efficiency of web service composition is extremely important for business organisations that provide services. However, most web testing only focuses on efficiency. This lacks the hybrid flexibility represented in Table 1. Furthermore, it does not focus on data accuracy or service defects. Simply focusing on efficiency is inadequate. In this study, functional and nonfunctional testing are implemented in a hybrid fashion to locate web services with minimum defects.

With regard to the optimal solution, harmony search (HS) algorithm [15] has many advantages compared with other metaheuristic algorithms [17], which imposes fewer mathematical requirements and does not require initial value settings of the decision variables. In particular, the HS algorithm uses stochastic random searches, with derivative information being also unnecessary and generates a new vector after considering all existing solutions. Many studies have modified the HS algorithm by dynamically updating the parameters and generating a new harmony search. For example, Improved harmony search algorithm called IHSA [21] is a modification been done in two parameters: pitch adjusting rate (PAR) and using bandwidth (BW) randomly. In addition, dynamic selection of BW and PAR parameters has been proposed [39]. Maximum and minimum values were replaced BW in HM process, and the PAR value was linearly decreased. In [20], a novel HS modification for both HMCR and PAR, dynamically in the improvisation process, was proposed. Al-Betar et al. [19] proposed a multipitch adjusting rate strategy to modify PAR. Sarvari and Zamanifar [22] proposed improvement in HS by statistical analysis, in which new harmony and BW is modified. Therefore, the proposed model improved harmony search (HS) algorithm and focused on the pitch adjustment rate (PAR) stage. Till date, HS algorithm used fixed values for PAR stages, and many researches modified HS for applications [18, 19, 40–44] where PAR is a very important parameter that can help in increasing the variations of the generated solutions by including more solutions in the search space of the optimal solution. This motivated current research in developing our proposed model to a new stage of PAR, called mutation-based HS approach.

## 3. Proposed Model

**Figure 1:**
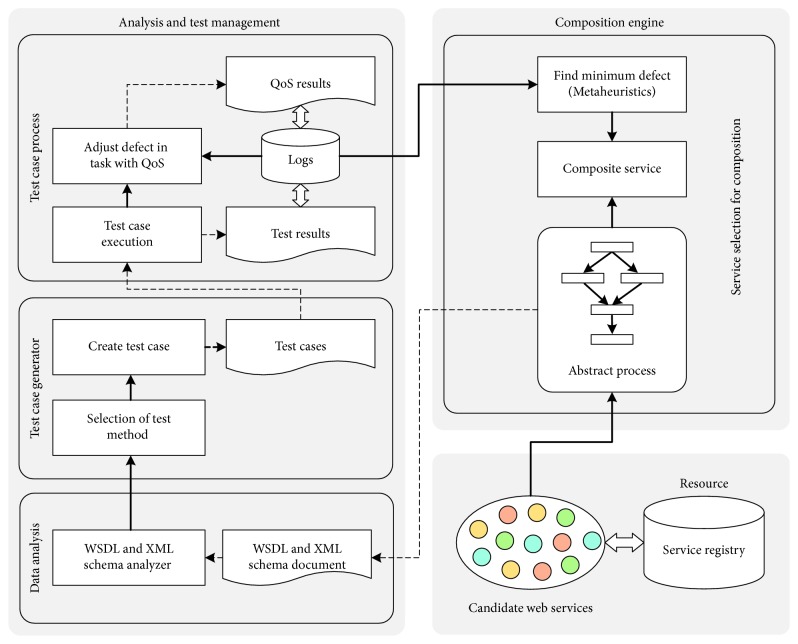
Overview of hybrid testing for web services.

**Figure 2:**
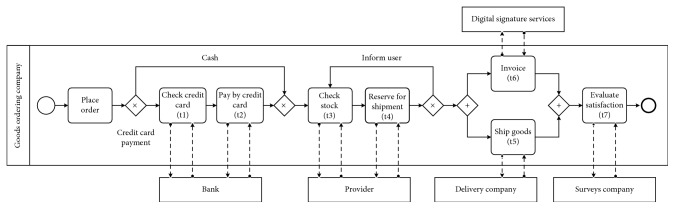
Example of goods ordering composite service.

This section presents a hybrid testing approach for the web service composition framework, as shown in Figure 1. Web service testing begins with the creation of a business process. Each process employs web services that are tested to provide services with minimum defects. Furthermore, QoS represents web service quality. For testing, a business process is created for a goods ordering service [34], as shown in Figure 2. It uses business process modelling notation, consisting of seven types of services and three representatives for each type. For example, one delivery company provides three types of services. The process begins with the ordering of goods, which can be paid in cash or by credit card. If credit card, the payment is verified and later accepted. Furthermore, the stock of goods is checked. The ordered goods will then be subtracted from the stock. If the ordered goods are out of stock, delivery will be delayed and annotated. When the goods are ready for delivery, it will be delivered to the customer with a digitally signed invoice. Finally, the customer's satisfaction will be monitored.

We present three processes for testing web service composition. First, we analyse the WSDL and XML schema definition (XSD) from the created business process. Test cases are designed using the equivalence class partitioning technique of functional testing. Finally, the developed test case is verified and measured to estimate the efficiency of the QoS for nonfunctional testing. After testing the web services, the one with minimum defects for composition is selected.

### 3.1. Data Analysis

**Figure 3:**
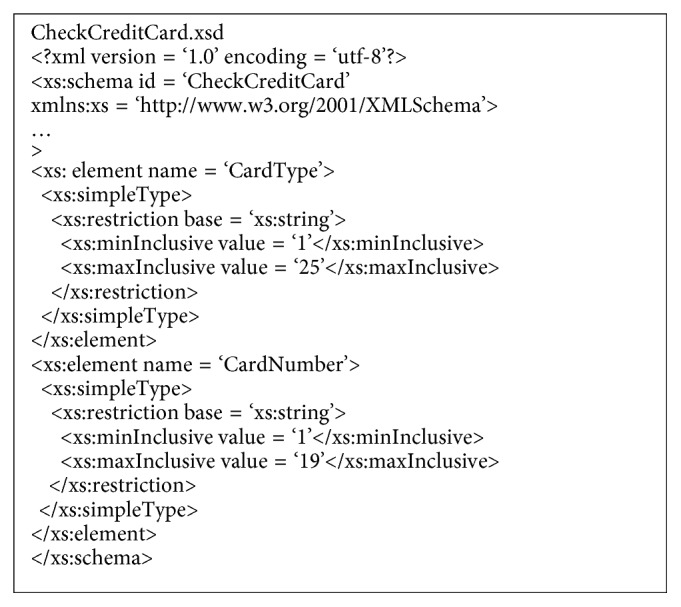
Example data type from XSD file.

The WSDL and XSD schema are analysed to find the operator, parameters, the data type of each parameter, and conditions for each data type. This is performed to set the conditions for testing (e.g., check credit card, Figure 3). Additionally, XSD data types are designed by the equivalence class partitioning method.

### 3.2. Test-Case Generator

The created test case from the schema analysis stage is thus implemented. Creating the data for the test case can be divided into two stages, as follows.

#### 3.2.1. Creating Equivalence Class Partitioning

**Figure 4:**
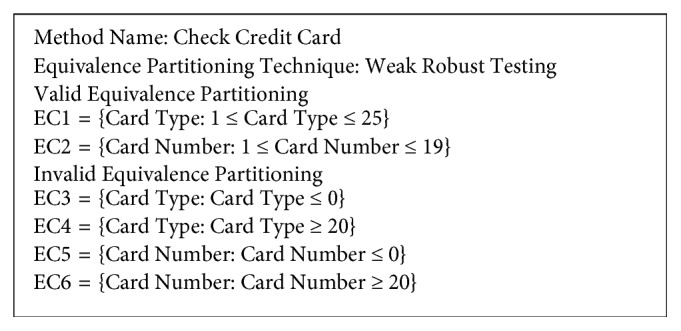
Example of equivalence class partitioning.

To create the equivalence class from WSDL and XSD of check credit card, the data are categorised into two groups: valid and invalid equivalence partitioning. This reduces the complexity of the data for testing, as shown in Figure 4.

#### 3.2.2. Creating Test Cases

The data, after partitioning through the equivalence class, are shown in Table 2. The test case (TC*n*) designs are stored as XML documents.

### 3.3. Test Case Process

#### 3.3.1. Test Case Execution

This is the process of evaluating defects found by the test case. The calculation results can be divided into three sections, as follows.

1. *Rate-of-Defect Detection (RDT*_*i*_). The RDT of a test case is calculated using the number of defects detected and number of test cases taken to find defects for each test case of web service, *i*

(1) $$\begin{array}{c} {\text{RDT}}_{i}=\frac{\text{number}\;\;\mathrm{of}\;\;\mathrm{defect}}{\text{number}\;\;\mathrm{of}\;\;\mathrm{testcase}}\times 10. \end{array}$$

(2) *Defect Impact (DI*_*i*_). For each defect severity value, defects have different impacts. The analysis of defects from the test case should be classified according to the severity level, as shown in Table 3. The severity value is calculated per the following equation:

(2) $$\begin{array}{c} S_{i}=\overset{t}{\underset{j=1}{\sum }}{\text{SV}}_{j}, \end{array}$$

where SV is the severity value of defect, *j*, and *t* is the number of defects identified by *S* of web service *i*.

The defect severity impact for each test case is defined as follows:

(3) $$\begin{array}{c} {\text{DI}}_{i}=\frac{S_{i}}{\max\left(S\right)}\times 10, \end{array}$$

where max(*S*) is the highest severity value of all test cases.

(3) *Test Case Weight (TCW*_*i*_). The TCW is the total sum of the two factors of web service *i* (i.e., RDT and DI). Mathematically, TCW_*i*_ can be computed by the following equation [45]:

(4) $$\begin{array}{c} {\text{TCW}}_{i}={\text{RDT}}_{i}+{\text{DI}}_{i}. \end{array}$$

The test results of the goods ordering case study are represented in Table 4. There are three service tasks, each having three web services available (i.e., WS1, WS2, and WS3). The bold value is the total sum of defect, which is calculated as TCW_*i*_.

#### 3.3.2. Adjusting Defect in Tasks with QoS

This process enhances the calculated service via functional testing, with an equal number of task defects (Table 5). This process is calculated by nonfunctional testing as QoS, which is divided into two parts of the calculation performance for each web service.

(1) *QoS Attribute.* The QoS describes the nonfunctional properties of the service. The QoS attributes of the candidate services defined in the related studies [28, 46, 47] are considered in this study.
(2) *QoS-Based Evaluation.* Some QoS attributes with higher or lesser values give better results separately. To adjust the process via normalisation, it is divided into three types: positive values (e.g. reputation (*R*)); negative values (e.g., response time (*T*) and execution cost (*C*)); and percentage values (e.g., availability (*A*)). The following equations are used to normalise the positive, negative, and percentage values, respectively
  (5) $$\begin{array}{c} {\text{QoS}}_{\text{reputation}}=\left(\frac{x-q^{\min}}{q^{\max}-q^{\min}}\right), \end{array}$$
  where QoS_reputation_ represents the value obtained from the conditional comparison; *x* is quality value of data which is required to be compared; *q*^min^ is the least quality value of data; and *q*^max^ is the maximum quality value of data
  (6) $$\begin{array}{c} {\text{QoS}}_{\text{response time}/\text{cost}}=\left(\frac{q^{\max}-x}{q^{\max}-q^{\min}}\right), \end{array}$$
  where QoS_response time/cost_ represents the value obtained via conditional comparison; *x* is the quality value of data which is required to be compared; *q*^min^ is the least quality value of data; and *q*^max^ is the maximum quality value of data
  (7) $$\begin{array}{c} {\text{QoS}}_{\text{availability}}=\frac{x}{100}, \end{array}$$
  where QoS_availability_ represents the value of conditioned comparison and *x* is quality value of data required for comparison.

After the score of each data is calculated and adjusted, the total score of the web service is calculated using Equation (8) to represent it as the weight of test results, instead of zero defects via the QoS weight score

(8) $$\begin{array}{c} {\text{TCW}}_{{\text{QoS}}_{i}}=1-\left(\frac{T_{i}+C_{i}+A_{i}+R_{i}}{\text{number}\;\;\mathrm{of}\;\;\mathrm{QoS}\;\;\mathrm{attributes}}\right), \end{array}$$

where TCW_QoS_*i*__ represents the total QoS_*i*_ weight score of the web service, *i*, which is less than satisfactory; *T*_*i*_ is the response time; *C*_*i*_ is the monetary value of the service; *A*_*i*_ is availability; and *R*_*i*_ is the reputation of web service *i*.

### 3.4. Service Selection for Composition by Mutation-Based Harmony Search

**Figure 5:**
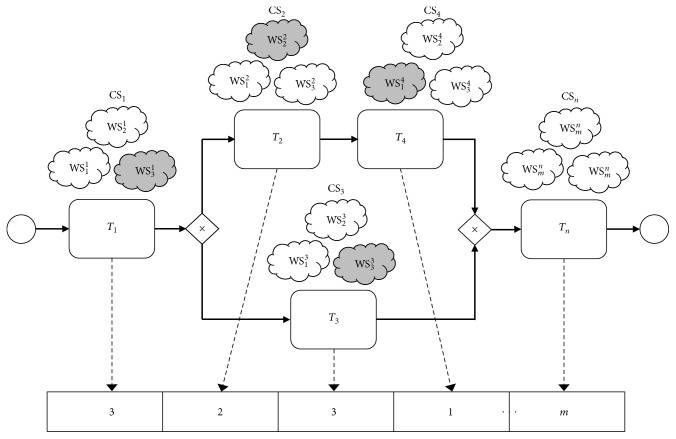
Service composition process.

This process provides a repository for the increasing task and various candidate services. For example, the candidate service from web service test results for each task is composed considering the business plan, as shown in Figure 2 and detailed in Figure 5. *T*_1_, *T*_2_,…, *T*_*n*_ are tasks that include the web service composition process, and CS_1_, CS_2_,…, CS_*n*_ are candidate service sets for the tasks, where the candidate service number of each set is *m*_*i*_, *i* ∈ {1,2,…, *t*}. The mathematical model of the web service composition can be described as follows:

(9) $$\begin{array}{c} \text{fitness}\;\;\mathrm{value:}\;\;\left\{\begin{array}{c} \min\;\;f\left(X_{i}\right),\\ f\left(X_{i}\right)=\overset{n}{\underset{i=1}{\sum }}{\text{TCW}}_{i}+{\text{TCW}}_{{\text{QoS}}_{i}}, \end{array}\right. \end{array}$$

where *X*_*i*_ represents a service selection scheme; *f*(*X*_*i*_) represents the fitness value; TCW_*i*_ is the weight of the test results by test case in functional testing; TCW_QoS_*i*__ is the weight of the test results by QoS in nonfunctional testing; and *n* is the number of tasks.

**Figure 6:**
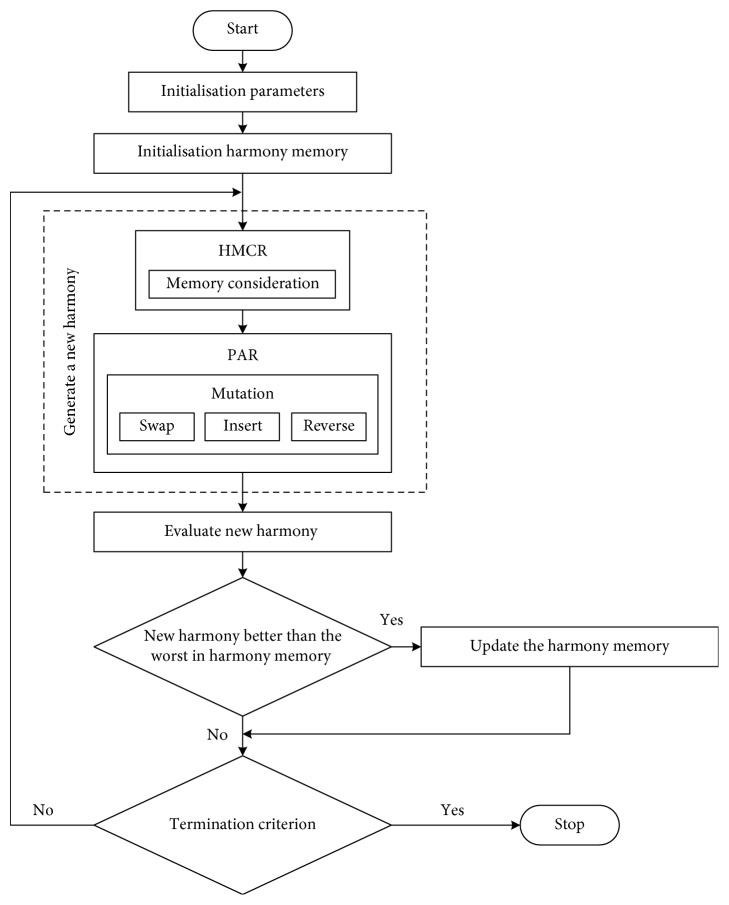
Mutation-based harmony search algorithm.

To solve the problem of web service composition, the HS algorithm is used to determine the fitness value while avoiding all possible compositions. However, the problem of the basic HS algorithm is continuous and cannot directly generate a candidate service for this problem. Therefore, we present a discrete variant of the HS algorithm and enhance a novel method of generating a new harmony pitch adjust rate (PAR) using mutation operations instead of random consideration. All the operators are summarised in Figure 6.

#### 3.4.1. Initialisation Parameters and Harmony Memory

First, we set the default parameter of the harmony memory size (HMS), harmony memory consideration rate (HMCR), PAR, and the termination criterion (e.g., maximum number of iterations). Furthermore, we randomly select from the candidate services of each task of the business plan. Those services are then initialised (i.e., HMS) and stored in the harmony memory (HM). Subsequently, we evaluate the fitness value using Equation (9). The population model is defined by Equation (10), where *X*^*i*^ is a candidate service and 1 ≤ *i* ≤ HMS

(10) $$\begin{array}{c} \text{HM}=\left[\left.\begin{array}{cccc} X_{1}^{1} & X_{2}^{1} & \cdots & X_{n}^{1}\\ X_{1}^{2} & X_{2}^{2} & \cdots & X_{n}^{2}\\ \vdots & \vdots & \cdots & \vdots \\ X_{1}^{\text{HMS}} & X_{2}^{\text{HMS}} & \cdots & X_{n}^{\text{HMS}} \end{array}\right|\begin{array}{c} f\left(X^{1}\right)\\ f\left(X^{2}\right)\\ \vdots \\ f\left(X^{\text{HMS}}\right) \end{array}\right]. \end{array}$$

#### 3.4.2. Generating a New Harmony

**Figure 7:**
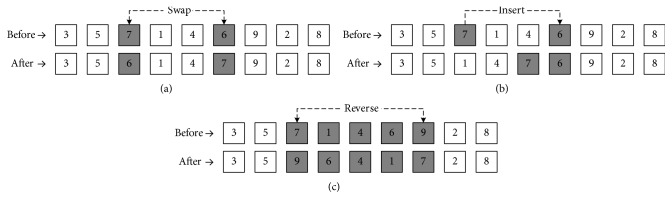
Mutation operators. (a) *Swap operator* (randomly selects the positions and swaps services in those positions). (b) *Insert operator* (randomly chooses two positions from a service and inserts the back one before the front). (c). Reverse operator (selects the sequence of the service of random length and then reverses the order).

At this stage, another harmony position is created, *X*^new^=[*X*_1_^new^, *X*_2_^new^,…, *X*_*n*_^new^], considering the HMCR. First, we randomise a digit between 0 and 1. If the value is less or equal to the HMCR, another position is created from memory. Otherwise, the new position is randomised in the set range. Furthermore, after obtaining every component of the memory consideration, we check whether the pitch should be adjusted. For randomising the digit between 0 and 1, mutation operations [48, 49] are used when the value is less than or equal to PAR for the nearest position. An example that explains these three operators are detailed in Figure 7.

#### 3.4.3. Updating the HM

At this stage, *X*^new^ is compared to *X*^worst^ in the HM. If a new harmony has a better fitness value than *X*^worst^, it is substituted at the new position with *X*^new^.

#### 3.4.4. Termination Criterion

Repeat the step of generating a new harmony and updating the HM until the stopping criterion (i.e., the number of iterations) has been satisfied with the best fitness value.

The pseudocode for the mutation-based HS algorithm is listed in Algorithm 1.

## 4. Experiment and Evaluation

This section describes the experiment and the results with a case study of web service composition. The proposed experimental framework is described as follows. First, the case study covers the testing activities performed on a sample business process model for servicing all composition plans of the candidate web service. Furthermore, experimentation settings and algorithm parameters are described. The aim of the experiment is to compare the performance of hybrid testing for the service composition with those using other metaheuristic algorithms for solving problems. Finally, the results are obtained from the case study of the framework for testing processes and composition recommendations.

### 4.1. Experimental Design

This section describes the methodology for the efficiency check to find web service composition defects via MBHS for the various business processes. For the first business model shown in Figure 2 [34], a goods ordering service consists of seven services: check credit card; pay by credit card; check stock; reserve for shipment; ship goods; invoice; and evaluate satisfaction. Each service includes representative service providers. In this experiment, each service has three tasks that show the defect results of service groups calculated from Table 6. The second business model concerns ticket reservation [50]. Considering the set of QoS attributes (i.e., cost, response time, availability, and reputation) and TCW, the QoS value ranges are randomly generated among 0 ≤ cost ≤ 100, 0 < response time ≤ 20 s, 60 ≤ availability ≤ 100, 0 ≤ reputation ≤ 10, and 0 ≤ TCW ≤ 10. The following experiment runs 20 times, and the results are averaged. The parameters used for each algorithm are described in the experiment. The service selection algorithm is realized using MATLAB 8.3.0.532 and is performed on a computer equipped with an Intel CPU, Core i7 @ 3.4 GHz, running on Windows 10 Pro 64 bit and 8-GB memory.

### 4.2. Feasibility and Scalability Analysis

**Figure 8:**
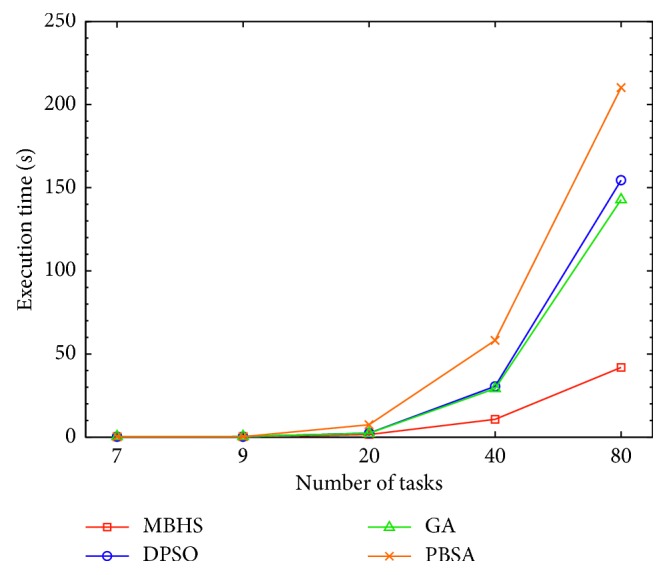
Performance with an increased number of tasks.

To analyse the feasibility using MBHS, the tasks are classified as 7, 9, 20, 40, and 80. Each task consists of 10 candidate services, which are compared by discrete PSO (DPSO) [51], GA, and population-based simulated annealing (PBSA) [52]. The response times of different tasks are evaluated and processed in a similar environment for estimating fitness value. The experimental results are presented in Figure 8, where the y axis indicates the execution time and the *x* axis indicates the number of tasks. The parameters used for each technique are described in Table 7.

According to Figure 8, there is an increase in tasks. MBHS uses less time compared to the other three algorithms. According to the experimental results, MBHS estimates the fitness value without considering time impacts, although the number of tasks increases.

### 4.3. Performance Analysis and Comparison

#### 4.3.1. Case Study I

To estimate the efficiency of MBHS for solving the problem of service selection with minimum defects, the defect results of the first business model (Table 6) are used. For this experiment, MBHS, DPSO, GA, and PBSA algorithms are applied to the service selection problem with minimum defects. The four algorithms are executed in the same environment, using the following parameters. The common parameters are initial population = 10 and iteration = 100. The MBHS settings are *N*_h_ = 10, HMCR = 0.9, and PAR = 0.1. The GA settings are Pc = 0.8, Pm = 0.1, and selector with elitism. The PBSA settings are initial population = 2, T0 = 100, alpha = 0.9, and nMove = 3.

**Figure 9:**
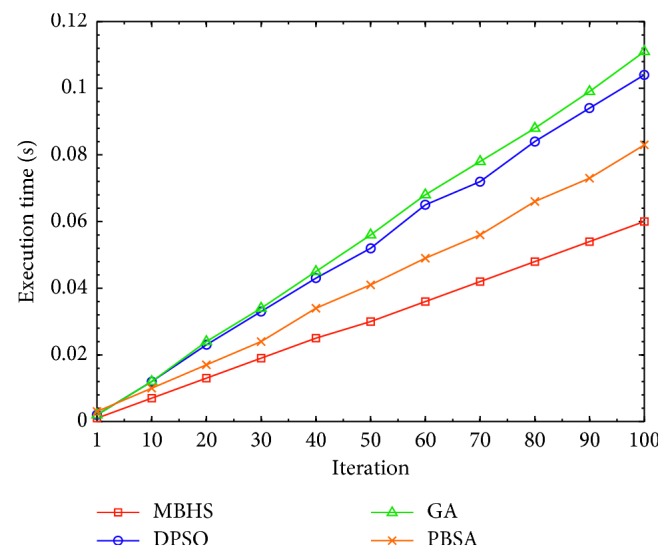
Execution time when the iteration increases.

**Figure 10:**
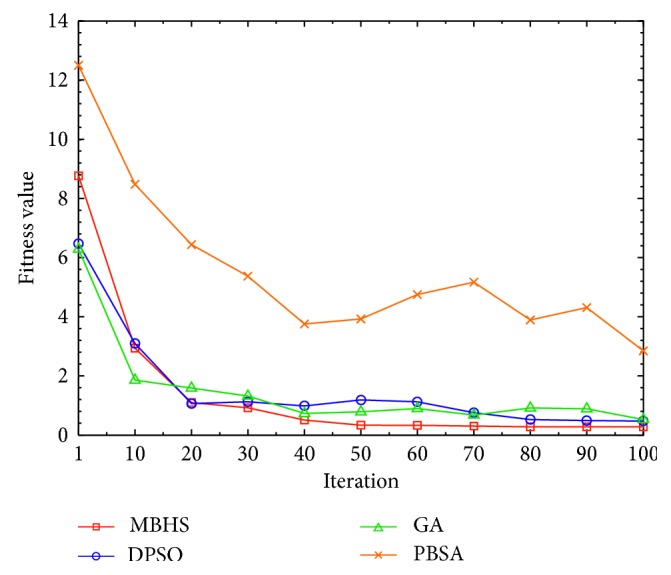
Performance of the algorithm when the iteration increases.

The four algorithms are executed, and the running times and fitness values are obtained by each algorithm and recorded. The experimental results are depicted in Figure 9, where the *y* axis indicates the execution time of the algorithm and the *x* axis indicates the iteration. In Figure 10, the *y* axis indicates the fitness value of the algorithm, and the *x* axis indicates the iteration. According to Figure 10, the MBHS algorithm can estimate the best fitness value within the same iteration, compared to the other three algorithms that estimate using more than 30 iterations. It also uses the least time for estimating the fitness value, as shown in Figure 9. The mean values obtained are presented in Table 8. The fitness value obtained by MBHS is 0.279 at the 80^th^ iteration. The PBSA algorithm has the worst fitness value, whereas the other algorithms have not yet obtained the solutions by the 100^th^ iteration. Thus, we conclude that MBHS is more efficient than the other algorithms.

#### 4.3.2. Case Study II

This experiment solves the service selection problem with minimum defects. The experiment is divided into tasks ∈ {20, 40} and candidate services ∈ {20, 60, 100} for service composition. To check the MBHS efficiency of this study, the DPSO, GA, and PBSA algorithms are used to solve the service composition defect. All algorithms are processed in the same environment for estimating the fitness value. For all the algorithms, the iteration is limited to 1,000.

**Figure 11:**
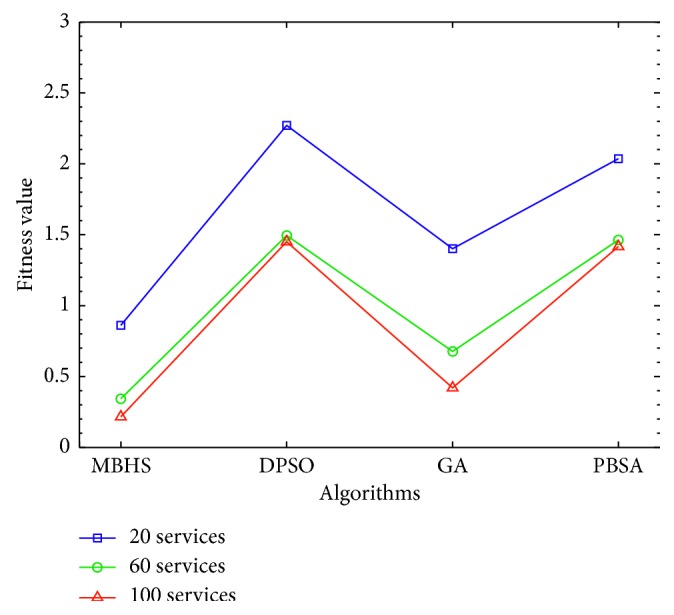
Performance comparisons for experiment I.

*(1) Experiment I*. The web service composition consists of 20 tasks and 20, 60, and 100 candidate services. The common parameter is the initial population = {30, 200, 400}. The MBHS settings are *N*_h_ = {7, 20, 35}, HMCR = 0.99, and PAR = 0.01. The GA settings are Pc = 0.8, Pm = 0.01, and selector with elitism. The PBSA settings are initial population = {20, 35}, T0 = 100, alpha = 0.99, and nMove = {5, 10}. The experiment results show that MBHS obtains the best fitness value, as shown in Figure 11, where the y axis indicates the fitness value of the four algorithms, and the *x* axis indicates the algorithm.

The execution time and fitness value estimated for the 20 tasks using the four algorithms are presented in Table 9, which show that MBHS is the quickest in estimating the fitness value at the 1,000^th^ iteration, whereas PBSA was the slowest. Furthermore, MBHS, with 20 candidate services, had a fitness value of 0.861, processed for 2.126 s. With 60 candidate services, it had a fitness value of 0.343, processed for 7.672 s. With 100 candidate services, it had a fitness value of 0.216, processed for 10.893 s. Compared to the other three algorithms, MBHS searched the best fitness value with the least time. According to this experiment, MBHS is more efficient than the other algorithms.

**Figure 12:**
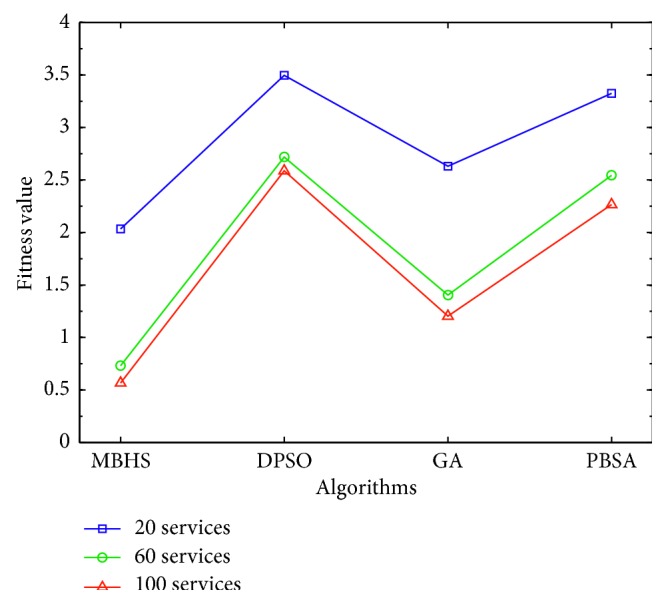
Performance comparisons for experiment II.

*(2) Experiment II*. The web service composition consists of 40 tasks and 20, 60, and 100 candidate services. The common parameter is initial population = {80, 300, 500}. The MBHS settings are *N*_h_ = {12, 25, 40}, HMCR = 0.99, and PAR = 0.001. The GA settings are Pc = 0.8, Pm = 0.01, and selector with elitism. The PBSA settings are initial population = {20, 45}, T0 = 100, alpha = 0.99, and nMove = {12, 20}. The experimental results show that MBHS has the best fitness value, as shown in Figure 12, where the y axis indicates the fitness value of the four algorithms, and the *x* axis indicates the algorithm.

The execution time and fitness value estimated for 40 tasks, using the four algorithms, are presented in Table 9. MBHS was the quickest in estimating the fitness value at the 1,000^th^ iteration, whereas PBSA was the slowest. Furthermore, MBHS, with 20 candidate services, had a fitness value of 2.034, processed for 7.346 s. With 60 candidate services, it had a fitness value of 0.731, processed for 12.367 s. With 100 candidate services, it had a fitness value of 0.566, processed for 15.010 s. Compared to the other three algorithms, MBHS searched for the best fitness value in least time. According to this experiment, MBHS is more efficient than the other algorithms.

## 5. Conclusion

This study proposed hybrid testing to overcome the problem of web service selection validity and reliability using MBHS to search for the minimum defect and provide the availability and accurate performance for web services in terms of functional and nonfunctional requirements. Furthermore, it is more complicated to enhance the service efficiency of a web service. However, doing so generally focuses on solving the problem without considering data accuracy. Thus, the proposed framework can be efficiently applied to detect defects. Additionally, it is most efficient for selecting web services for composing. To compare web service selection using different algorithms, a test case for testing the QoS, based on the functional requirement, was required. Furthermore, it required nonfunctional requirements (e.g., response time, cost, availability, and reputation) for testing web services. The MBHS algorithm combined hybrid testing and helped select various web services per business requirements.

## Figures and Tables

**Algorithm 1:**
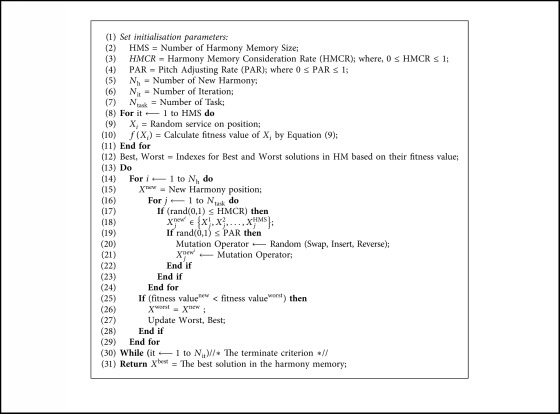
Mutation-based HS.

**Table 1:** Comparisons of proposed model and other works.

Related works	Objective			System under test
	Functional testing	Nonfunctional testing	Algorithms	Service composition
Mardukhi et al. [29]		X	GA	X
Liu et al. [30]		X	GA	X
Fan et al. [33]		X	PSO + SA	X
Parejo et al. [34]		X	GRASP + PR	X
Yu et al. [35]		X	GRASP + ACO	X
Mao et al. [36]		X	PSO	X
Fanjiang et al. [37]		X	GP	X
Liu et al. [38]		X	SLO	X
Proposed model	X	X	MBHS	X

**Table 2:** Test case designs with equivalence class using weak robust method.

Test case	EC ID	Card type	Card number	Expected results
TC1	EC1, EC2	VISA	4024007146100680	The number is valid!
TC2	EC1, EC5	VISA	−1145422424242	The number is not valid!
TC3	EC1, EC6	Master Card	54872885221971100000	The number is not valid!

**Table 3:** Severity value of defect impact.

Level of severity	Severity value (SV)
Critical	4
High	3
Medium	2
Low	1

**Table 4:** Test case execution results of goods ordering (functional testing).

Task of service	Test results of test case weight (TCW_*i*_)		
	WS1	WS2	WS3
T1: check credit card	8.333	16.666	0.000
T2: pay by credit card	0.000	0.000	0.000
T3: check stock	0.000	0.000	0.000
T4: reserve for shipment	4.231	8.415	0.000
T5: ship goods	0.000	8.365	4.156
T6: invoice	12.565	10.365	0.000
T7: evaluate satisfaction	0.000	0.000	0.000

**Table 5:** Typical QoS attributes of component services.

QoS attribute	Definition	Notation
Response time (*T*)	The elapse time between a request and a response	*T*=*T*_1_ − *T*_0_
Execution cost (*C*)	The cost associated with the invocation of a service	Monetary value of the service
Availability (*A*)	The probability that a service is accessible and ready for immediate use	*A*=(uptime/(uptime+downtime)) × 100
Reputation (*R*)	The measurement of a service's trustworthiness	Calculated based on the experiences of the users

**Table 6:** Overall testing execution results of goods ordering (functional and nonfunctional testing).

Task of service	Test results of test case weight (TCW_*i*_) and QoS weight (TCW_QoS_*i*__)		
	WS1	WS2	WS3
T1: check credit card	8.333	16.666	0.000
T2: pay by credit card	0.567	0.454	0.089
T3: check stock	0.125	0.756	0.424
T4: reserve for shipment	4.231	8.415	0.000
T5: ship goods	0.000	8.365	4.156
T6: invoice	12.565	10.365	0.000
T7: evaluate satisfaction	0.847	0.065	0.147

**Table 7:** Parameters of four algorithms in the feasibility and scalability analysis.

Algorithm	Population size	Iteration	Value of other parameters
MBHS	{20, 30, 200, 500}	{200, 1000, 2000, 4000}	Number of new harmonies: *N*_h_ = {5, 10}
			HMCR = {0.90, 0.99}
			PAR = {0.01, 0.1}
DPSO	{20, 30, 200, 500}	{200, 1000, 2000, 4000}	—
GA	{20, 30, 200, 500}	{200, 1000, 2000, 4000}	Probability of crossover: Pc = 0.8
			Probability of mutation: Pm = {0.01, 0.1}
			Selector: Elitism
PBSA	{2, 10, 15, 20}	{200, 1000, 2000, 4000}	Initial temperature: T0 = 100
			Cooling rate: Alpha = {0.9, 0.99}
			Number of neighbor for each member: nMove = {3, 10, 15, 25}

**Table 8:** Iteration, mean fitness, and average time of the four algorithms.

Iteration	MBHS (proposed algorithm)		DPSO		GA		PBSA	
	Fitness	Time (s)	Fitness	Time (s)	Fitness	Time (s)	Fitness	Time (s)
1	8.763	0.001	6.473	0.002	6.286	0.002	12.500	0.003
10	2.934	0.007	3.096	0.012	1.856	0.012	8.475	0.010
20	1.097	0.013	1.063	0.023	1.587	0.024	6.436	0.017
30	0.920	0.019	0.983	0.033	1.319	0.034	5.372	0.024
40	0.506	0.025	1.185	0.043	0.730	0.045	3.753	0.034
50	0.333	0.030	1.124	0.052	0.784	0.056	3.924	0.041
60	0.329	0.037	0.755	0.065	0.894	0.068	4.751	0.049
70	0.301	0.042	0.522	0.072	0.675	0.078	5.170	0.056
80	0.279	0.048	0.487	0.084	0.915	0.088	3.892	0.066
90	0.279	0.054	0.678	0.094	0.888	0.099	4.311	0.073
100	0.279	0.060	0.472	0.104	0.523	0.111	2.847	0.083

**Table 9:** Average of fitness value and average time for the four algorithms.

Task	Candidate services	MBHS		DPSO		GA		PBSA	
		Fitness	Time (s)	Fitness	Time (s)	Fitness	Time (s)	Fitness	Time (s)
20	20	0.861	2.126	2.271	2.447	1.401	2.388	2.036	5.306
	60	0.343	7.672	1.495	14.776	0.677	12.220	1.463	18.829
	100	0.216	10.893	1.450	29.406	0.420	23.134	1.417	42.251

40	20	2.034	7.346	3.496	15.218	2.631	12.662	3.325	18.604
	60	0.731	12.367	2.718	30.210	1.404	25.386	2.544	44.988
	100	0.566	15.010	2.588	38.206	1.203	34.096	2.266	56.875

## Data Availability

The 7 tasks of service data used to support the findings of this study are available from the cited paper [34]. The 9 tasks of service data used to support the findings of this study are available from the corresponding author upon request. And 20, 40, and 80 tasks of service data used to support the findings of this study are available from simulation in experiment upon request.
